# Inspection and maintenance of industrial infrastructure with autonomous underwater robots

**DOI:** 10.3389/frobt.2023.1240276

**Published:** 2023-08-25

**Authors:** Franka Nauert, Peter Kampmann

**Affiliations:** ^1^ Subsea Solutions, ROSEN Creation Center GmbH, Bremen, Germany; ^2^ Faculty of Technology, University of Bielefeld, Bielefeld, Germany

**Keywords:** autonomous underwater intervention, intervention AUV, subsea inspection and maintenance, non-destructive testing, pipeline inspection, underwater cleaning, underwater welding

## Abstract

Underwater infrastructure, such as pipelines, requires regular inspection and maintenance including cleaning, welding of defects and valve-turning or hot-stabbing. At the moment, these tasks are mostly performed by divers and Remotely Operated Vehicles (ROVs) but the use of intervention Autonomous Underwater Vehicles (intervention-AUVs) can greatly reduce operation time, risk, and cost. However, autonomous underwater manipulation has not yet reached a high technological readiness and is an intensively researched topic. This review identifies key requirements based on necessary inspection and maintenance methods, linking them to the current technology and deriving major challenges which need to be addressed in development. These include the handling of tools, where a separation between handheld and mounted tools is detected in already employed underwater intervention vehicles such as the Sabertooth by Saab Seaeye or the Aquanaut by Nauticus robotics, two vehicles capable of semi-autonomous intervention. The main challenge identified concerns high level autonomy, i.e., the process of decision-making. This process includes detecting the correct point of interest, maximizing the workspace of the manipulator, planning the manipulation considering required forces, and monitoring the progress to allow for corrections and high quality results. In order to overcome these issues, reliable close range sensing and precise end point navigation is needed. By identifying these persisting challenges, the paper provides inspiration for further development directions in the field of autonomous underwater intervention.

## 1 Introduction

Underwater structures, such as pipelines, jacket-type supporting structures of platforms or wind turbines are exposed to many hazards. Their structural integrity decreases due to corrosion and they are exposed to strong waves and currents, depending on their installation site (Kang et al., 2013). Additionally, marine growth attaches to the structures, making them heavier and increasing their diameter, so that waves and currents have an even stronger impact (Pedersen et al., 2022). All of these factors diminish the stability of the structure, increasing the risk of a failure or leakage which can pose severe threads to the environment.

However, regular inspection of the structures helps to minimize the risk of failure as small defects are detected early and can be remediated in time. There are several inspection methods, which can be classified depending on the level of detail that is desired. General visual inspection (GVI) for example, is used to identify large defects and does not require cleaning of the surface. Detailed Visual Inspection (DVI), on the other hand, requires cleaning to a certain extent and Close visual inspection (CVI) is used to identify visible corrosion or pitting on a clean structure, from which the marine growth has been completely removed (Outa et al., 2015). This paper will focus on CVI and other contact based inspection methods, since these provide more detailed information about the structure and are, therefore, mandatory for the maintenance of its integrity.

Apart from inspection, intervention is another important aspect of maintaining underwater structures. This comprises the turning of valves at oil and gas wells in order to control the production flow, as well as the welding of defects. Another part of intervention is cleaning which closely intersects with inspection since many inspection methods require direct contact to the structure.

Nowadays, the inspection and maintenance of underwater infrastructure is mostly performed by trained divers or remotely operated underwater vehicles (ROVs). However, both have their drawbacks: the divers face many safety hazards, and are limited in their operating depth and time, while the ROVs need a large surface vessel capable of Dynamic Positioning (DP) with experienced operators and are limited in their maneuverability due to the umbilical connecting them to the support vessel. Autonomous Underwater Vehicles (AUVs) do not have said umbilical, and they do not need an extensive crew of operators, which makes them a good alternative to ROVs. Most common AUVs are only used for GVI, but there is a rising interest in Intervention AUVs (I-AUVs) which possess one or two manipulator arms and the appropriate tools to perform contact based inspection and basic maintenance tasks like valve turning or hot stab operations (Palomeras et al., 2014; Simetti et al., 2018; Pi et al., 2021; Christensen et al., 2022).

Currently, AUVs are employed for survey and inspection tasks in several fields such as marine geology (Wynn et al., 2014), archeology (Bingham et al., 2010), the tracking of hydrocarbon plumes (Camilli et al., 2010) and visual inspection of underwater infrastructure such as hydroelectric dams or oil and gas infrastructure (Ridao et al., 2010; Gilmour et al., 2012). However, even though the level of autonomy needed for survey tasks has reached a high technological readiness, the performance of intervention tasks is still an intensively researched topic. Depending on how one wants to tackle this issue, several challenges need to be overcome. Identifying the location of operation with onboard sensors under varying environmental conditions, choosing optimal points of contact for intervention or contact based measurements and planning and executing the intervention task itself, just to name a few.

This paper aims to derive a set of capabilities and challenges for I-AUVs from common inspection and maintenance methods and to discuss whether the current state of the art can meet these requirements. There are several other surveys around this topic, for example, an extensive review which focuses on inspection and monitoring systems for subsea pipelines (Ho et al., 2020) in general while not looking at potential future applications of autonomous robotic technologies. Meanwhile, recent developments in autonomous underwater intervention with a focus on control systems are demonstrated in (Simetti, 2020), where examples of successful free floating manipulation are presented. Similar reviews provide information about recent developments in manipulator or sensor technology (Cong et al., 2021; Morgan et al., 2022). Compared to the review papers presented here, this paper seeks to create a link between recent technological developments and how they can be used for existing inspection and maintenance methods and to identify gaps which still need to be covered by ROVs or human divers.

The further structure of this paper is as follows: First, frequently used inspection and maintenance (IM) methods are presented. Next, a set of requirements is derived and recent advances in AUV and manipulator technology are reviewed in correspondence with their potential use for the IM methods. Challenges, which still persist, are then discussed in the next section and finally, a potential outlook is proposed.

## 2 Inspection methods

Inspection of industrial structures minimizes the risk of failure, since small defects can be detected and remediated before they pose severe threads to the integrity of the structure. There exist several inspection methods employing different physical principles. Visual inspection is a very common method since it permits to gain a quick overview of the structure. However, it is hard to gain quantitative information of the integrity of the structure when using visual inspection and internal defects can not be detected. Therefore, other Non-Destructive Testing (NDT) have been developed using ultrasound or electromagnetic fields to penetrate the structure and determine internal flaws. Other methods like Cathodic Protection (CP) seek to actively prevent defects, creating a need for regular inspection, to see if the structure is still protected. This section aims to explain the principles of common inspection methods and to describe how they are currently performed, highlighting that most of them are not yet employed by AUVs.

### 2.1 Visual inspection

Visual inspection is differentiated into general, detailed and close visual inspection, depending on the accuracy with which the structure is inspected. This terminology is defined by Outa et al. (Outa et al., 2015) and used similarly by a number of industries including pipeline inspection (DIN-Normenausschuss Materialprüfung, 2016) and aircraft maintenance (Mainblades, 2023). General Visual Inspection (GVI) gives a broad overview of major defects, visible from afar without the need to remove marine growth. For Detailed Visual Inspection (DVI), however, the structure must be cleaned to a certain extent and Close Visual Inspection (CVI) needs a completely cleaned structure to observe corrosion pitting and inspect welds (Outa et al., 2015). Detailed quantitative differences between GVI, DVI and CVI could not be found in the literature, but EN 13018 (Det Norske Veritas, 2021) differentiates between local visual inspection (similar to DVI and CVI) and general visual inspection (corresponding to GVI). Local visual inspection is carried out at a distance below 600 mm at an angle below 30^*o*^ and an illumination of minimum 500 lx while general visual inspection is carried out at distances larger than 600 mm with an illumination of minimum 160 lx. Visual inspection in shallow depths is typically performed by divers and to perform visual inspections remotely, ROVs with attached cameras can be used. For GVI, AUVs have already been employed, as no interaction with the structure is needed. DVI and CVI, however, require cleaning of the structure, which is so far a demanding task for AUVs.

### 2.2 Acoustic methods

Acoustic methods use the propagation and reflection of ultrasonic waves sent into the structure to determine inner flaws. There are different acoustic methods varying in terms of accuracy, measured area and employed hardware.

**FIGURE 1:**
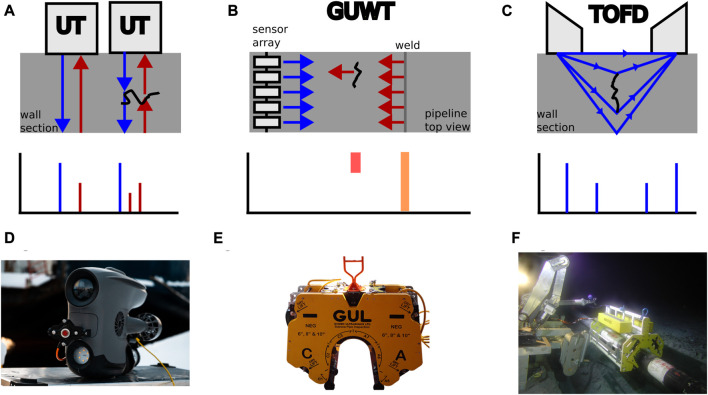
The principles of different acoustic testing methods are shown in **(A)**, **(B)** and **(C)**. Emitted sound waves are blue, reflected sound waves red. **(D)**, **(E)** and **(F)** show tools for the different inspection methods. **(D)** Shows a fix mounted UT thickness gauge (courtesy of Blueye Robotics), **(E)** shows a ROV operated GUWT tool (courtesy of Guided Ultrasonic Ltd.) and **(F)** shows a ROV deployed crawler with incorporated TOFD scanner (courtesy of Sonomatic).

Ultrasonic Testing (UT) is one of the most common external pipeline inspection methods. The sensor consists of a transceiver which sends an ultrasonic wave into the structure. This wave is reflected by physical boundaries such as the other side of the wall or cracks, which provide a double echo since only part of the wave is reflected at the crack (see Figure 1A). It can be used as a single sensor or as an array of sensors (Phased UT) which then provides information for a cross-section. It has a high resolution and can inspect internal as well as external effects, but it requires a clean surface, in some cases even coatings have to be removed, since the piezoelectric sensors need to be in direct contact with the structure. Additionally, a stable contact during the measurement is required and UT can only inspect a small area at once (Ho et al., 2020). Phased and single spot UT sensors can be used together in ultrasonic scanners which can be incorporated in small crawlers that are deployed by ROVs (Jeppesen et al., 2005). Additionally, there exist UT thickness gauges meant to be mounted on ROVs (see Figure 1D) (Antony Jacob et al., 2021; CYGNUS, 2023) as well as diver held UT inspection tools (Oceanscan, 2023a).

Guided Ultrasonic Wave Testing (GUWT) can inspect larger areas by using lower frequencies than UT by sending ultrasonic waves parallel to the structure. In case of defects such as wall thinning or weld imperfections, a part of the wave is reflected back towards the sensor (see Figure 1B). Pipeline inspection is done by attaching an array of sensors around the pipeline. This permits to inspect long sections of the pipeline (up to 100 m) at once, but at a lower resolution, since the low frequency waves with long wavelengths do not interact with small cracks (Cawley et al., 2003; Ho et al., 2020). The level of cleaning required to perform GUWT depends on the sensor technology used. Piezoelectric sensors require a clean structure similar to UT, while electromagnetic acoustic transducers (EMATs) work over non-metallic debris on the structure. They are, however sensitive to magnetic fields and require more power than piezoelectric sensors (Ho et al., 2020; Jacques et al., 2020). The array can be deployed by a diver or ROV and requires an actuation in order to close around the pipeline (see Figure 1E). The ROV mountable tool uses hydraulic actuation to close, while the diver operated tool has a mechanical clamping mechanism (Ultrasonics Ltd, 2023). These tools only require minimal surface preparation.

In contrast to UT and GUWT, which analyze the reflection of the ultrasonic wave, the Time Of Flight Diffraction method (TOFD) uses two separated transducer and receiver probes to analyze the diffraction of the sound wave in order to detect cracks and inspect welds. A wide beam is projected into the structure and the receiver will receive two major signals, one from waves travelling along the surface of the structure and one from the reflection of the wave at the bottom of the structure (see Figure 1C). In presence of a crack, the diffraction of the wave at the edges of the defect will also be measured (Outa et al., 2015; Ho et al., 2020). As shown in Figure 1F, TOFD sensors can be incorporated in UT scanners which are deployed by ROVs (Jeppesen et al., 2005).

Since these inspection methods have not yet been adapted for autonomous inspection, no examples of AUVs performing acoustic inspection could be found. In Section 4, requirements for AUVs will be derived, based on the challenges posed by close range inspection.

### 2.3 Electromagnetic methods

Electromagnetic methods induce a current in the structure, which in turn induces a magnetic field that can be measured. Defects in the structure alter the current and therefore be detected in the magnetic field. The electromagnetic methods vary mostly in the way the current is induced.

**FIGURE 2:**
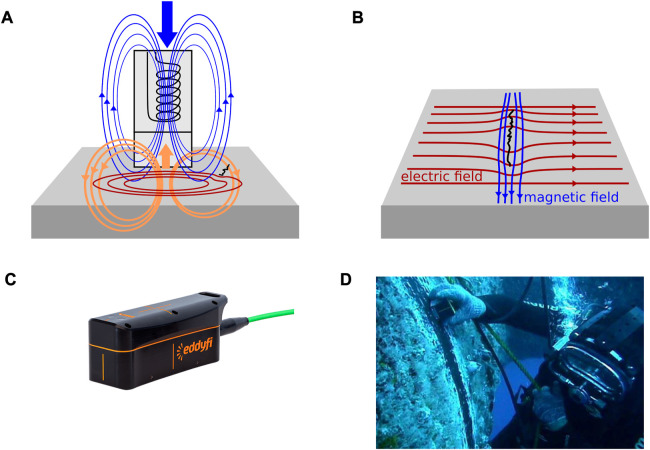
Principals of different electromagnetic inspection methods. EC is displayed in **(A)**, the generated magnetic field is shown in blue, the induced eddy currents in red and the measured magnetic field in orange. In **(B)** ACFM is shown. Tools for EC and ACFM measurements are shown in **(C)** and **(D)** (courtesy of Eddify Technologies).

The Eddy Current method (EC) uses changes in a magnetic field, which passes orthogonal to the structure to induce circular electric currents in the structure. The magnetic field is generated by passing an alternating current or a pulsed current through a coil. The lower the frequency of the alternating current, the higher the penetration depth. However, low frequencies need more energy. By passing a pulse through a coil, several frequencies are emitted simultaneously, and larger penetration depth can be achieved with less energy. This method is called Pulsed Eddy Current (PEC). The induced magnetic field generates circular eddy currents in the structure which show irregularities in the presence of defects. These eddy currents in turn generate an opposing magnetic field which reflects these irregularities and is measured by a sensor (Ho et al., 2020) (see Figure 2A). Another method to detect metal loss on the far side is the Magnetic Eddy Current (MEC). Therefore, an additional magnetic field, flowing through the entire depth of the structure is generated. Changes on the far side will affect this bias field, which in turns affects the field measured by the EC sensors. MEC sensors can be incorporated in crawlers, as shown in the MEC Combi Crawler by (Reber et al., 2016). Another example of EC performed by ROVs is shown in (Antony Jacob et al., 2021), where a PEC Probe is mounted to a small ROV in order to measure wall thickness through a layer of marine growth. An example of such a probe is shown in Figure 2C.

Another electromagnetic inspection method is the Alternating Current Field Measurement (ACFM) where a local uniform, alternating current is passed through the surface. This current flows close to the surface and is associated with an orthogonal magnetic field. Cracks in the surface can be measured through disturbances is the magnetic field, the deeper the crack, the more severe the disturbance (Raine and Lugg, 1999) (see Figure 2B). Different possibilities to use ACFM Tools with ROVs are shown in (Lugg, 2011) There are standard probes which need to be grasped by a manipulator (see Figure 2D) or probes mounted directly on the end effector, thereby providing more stability. Another method is the deployment of automated scanners, which can be attached to the inspection site.

Electromagnetic inspection requires a stable contact with the surface for the duration of the measurement, which makes it challenging for AUVs. However, since there are already autonomous scanners, the transition to a fully autonomous inspection vehicle is quite probable.

### 2.4 Cathodic protection

Corrosion is an electrochemical process, where an anode gets oxidized, freeing electrons which flow to a cathode. In order to protect a structure from corroding, a negative voltage can be applied to it is surface, making it a cathode. This can be achieved by attaching a less noble metal to the structure which serves as a sacrificial anode that must be replaced at some point (Outa et al., 2015). Another method, which is preferably used for larger structures, is the Impressed Current Cathodic Protection (ICCP). Here, the anode is connected to a DC power source, which allows it to cathodize the structure without deteriorating (Popov et al., 2005). Since sacrificial anodes have no need of an external power source and are easier to install, they are the preferred method of protection on offshore structures (Okyere, 2019). In order to measure the effectiveness of the installed CP (i.e., if the anodes need to be changed) and to determine the risk of corrosion, CP measurement probes are employed by ROVs or Divers. They usually consist of a contact and a reference electrode in order to measure the potential of the structure. There are Probes equipped with a handle to be grasped by a manipulator (Subsea TechnologyRentals, 2023) and probes that need to be mounted directly onto the ROV or manipulator (Oceanscan, 2023b). However, Kowalczyk et al. (Kowalczyk et al., 2019) have developed a non-contact CP measurement method for pipelines, that has successfully been carried out by an AUV at a distance of 10 m to the pipeline.

## 3 Manipulation and maintenance methods

After the inspection of underwater structures, potentially detected defects must be remediated in order to maintain the integrity of the structure. This maintenance includes the welding of cracks, or the replacement of sacrificial anodes at the end of their lifespan. Cleaning can also be considered as a form of maintenance, since it considerably reduces the drag executed on the structure by currents and waves. In contrast to other maintenance methods, cleaning is often executed before the inspection, since many NDT methods (like UT or TOFD) require a close contact to the surface. In addition to maintenance, there are other manipulation tasks, that need to be performed underwater. Especially at oil and gas mining sites, some intervention is needed to adjust the product flow. This can be achieved by manipulating an underwater christmas tree (a hub of valves and connections at an oil or gas well), which includes the turning of valves or the creation of a hydraulic connection between the ROV and the tree by hot stabbing. This section aims to describe some of the most common maintenance and manipulation methods and to highlight how they are performed.

### 3.1 Cleaning

The attachment of marine growth on industrial structures poses a thread to their integrity, since it increases diameter and weight and results in higher loads induced by waves. The largest amount of marine growth is situated in the splash zone, where light is abundant. The splash zone is heavily influenced by waves and wind, and provides different challenges than the quiet depth, which might influence the robot design (Tecchio et al., 2021). Additionally, the amount of marine growth varies with the location, since many marine fouling organisms grow better in warmer waters (Lord, 2017). However, even in the North Sea, fouling organisms can decrease the minimum tension of mooring lines by up to 62% thereby increasing the risk of snap loading considerably, which shows the importance of cleaning (Wright et al., 2016). Additionally, many inspection methods, such as UT, TOFD or conventional CP measurements, require a direct contact to the surface and, thus, the removal of bio-fouling.

Cleaning is therefore needed on all underwater structures. Oil and gas platforms typically have bio-fouling removed periodically, while offshore wind platforms often oversize the foundations in order to cope with the increased hydrodynamic load and only need cleaning for inspection tasks (Pedersen et al., 2022). For ships, marine growth produces an even greater challenge, since the increased hydrodynamic load results in an increased propulsive load and, therefore, higher fuel consumption. They are often coated with antifouling substances, but this protection is not durable and frequent cleaning is required (Song and Cui, 2020). An additional problem has been highlighted recently, as a cruise ship was denied entry to New Zealand and Australian ports due to foreign bio-fouling on its hull that posed a bio-security risk (Viking Orion, 2023).

There are several tools to perform cleaning which use different principles. Rotary brushes or barnacle cutters mechanically remove marine organisms by scraping them off the surface. These tools require permanent contact to the surface, and they can be harmful on protruding structures such as welds or rivets. This risk can be reduced using contact-less pressure based tools, like water jets or cavitation blasters (Floerl et al., 2010). Water jets rely on the impact of the water jet to clean the hull, while cavitation blasters function like water jets but have specially designed nozzles, that introduce tiny bubbles on the stream which produce extra stress upon rupturing (Kalumuck et al., 1997). Even though there exist balanced tools, pushing the water in both directions to stabilize themselves (DiveWise Equipment, 2023), most tools are simple and constantly pushed away from the surface, requiring a good adhesion mechanism or strong thrusters to keep a stable distance.

Other technologies, like ultrasonic cleaning or laser cleaning permit a gentle but thorough cleaning without generating repulsive forces. Ultrasonic cleaning uses multiple frequencies to generate an alternating pattern of positive and negative pressure waves. This induces and implodes tiny bubbles creating local stress at the implosion, which has a cleaning effect (Awad et al., 2010). Laser cleaning can either be focused directly on the surface, vaporizing the grime (Chen et al., 2012), or parallel to it, emitting shock-waves that cause cavitation bubbles which attach to the surface and clean it (Song et al., 2004).

For mechanical, hydrodynamic and ultrasonic cleaning, tools are available for ROVs (Yan et al., 2019; Nandha et al., 2021; Mai et al., 2022) and divers (Courson and Shelbourne, 2000; Blue Phoenix, 2023; DiveWise Equipment, 2023) alike, even though ROVs were more commonly employed in the last decades, since their technology is mature, and they are safer than divers (Mai et al., 2017; Pedersen et al., 2022). Laser cleaning is probably only performed by ROVs, since no commercially available tools for divers could be found.

A major drawback of all these tools is their requirement of power. According to Mai et al. (Mai et al., 2022) most effective cleaning methods require over 10 kW, while cleaning operations might last up to 10 h. This, of course, makes it difficult to incorporate them in AUVs, which only have a limited power amount provided by their battery. Another challenge for AUVs, depending on the cleaning method, would be to keep a stable distance between the tool and the Wall. This has already been addressed for ROVs, where several attaching mechanisms using magnets, negative pressure or thrusters have been developed (Song and Cui, 2020). However, these mechanisms would have to be reevaluated for the employment on AUVs.

### 3.2 Welding

As mentioned above, cracks detected by inspection need to be remediated in time, in order to avoid critical failure of the structure. For smaller cracks in jacket type structures, grinding, or hole drilling can stop the propagation, but a weakening of the structure still persists. Larger cracks, on the other hand, need to be welded in order to reinstate the original strength of the structure. Additionally, underwater welding permits the attachment of strengthening plates, or sacrificial anodes to the structure, thereby increasing its integrity (Sharp and Ersdal, 2021). The technique is, therefore, used for repairs on subsea pipelines, ships, nuclear power plants and other metallic components (Surojo et al., 2020).

Underwater welding can be distinguished into two main categories: dry welding and wet welding (Paton, 1998). Dry welding is accomplished by encapsulating the working area with an air or gas filled tank, so that welding can be performed in conditions similar to those on land. This yields good results but is very expensive and time-consuming. Wet welding, in contrast, is performed directly in the water, which makes it faster, cheaper and more versatile as it can reach complex geometries. However, there are several difficulties associated with wet welding, namely, the formation of hydrogen and oxygen in gas pockets, which can lead to an explosion, the formation of pores in the weld and the faster cooling rates which decrease the ductility of the metal and increase the risk of cold cracking (Majumdar, 2006; Łabanowski et al., 2008). These difficulties have led to a low usage rate of wet underwater welding, even though there are advanced welding methods which can overcome these challenges (Majumdar, 2006). This section will focus on wet welding, since it can more readily be employed by Unmanned Underwater Vehicles (UUVs), including both ROV and AUV, than dry welding.

Wet welding can be performed using several methods. Conventional welding methods use an electrical arc to melt the metal. Shielded Metal Arc Welding (SMAW) and Flux Cored Arc Welding (FCAW) are the preferred methods for underwater arc welding, currently employed by diver-welders for repairs (Surojo et al., 2020). Other methods include laser welding, where the metal is heated with a laser beam and friction welding which induces coalescence of to materials by rubbing them together. These methods will be explained below, in order to provide context and thought impulses for the development of robotic applications.

#### 3.2.1 Shielded Metal Arc Welding (SMAW)

**FIGURE 3:**
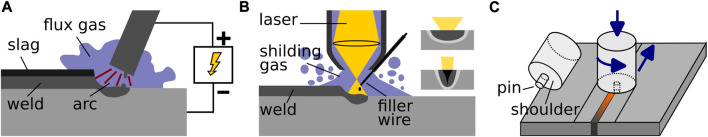
The principles of different welding methods are shown above. Arc welding at the example of SMAW is depicted in **(A)**. In **(B)** a schematic of local cavity laser welding is depicted, showing the nozzle and shielding gas. On the right the different welding modes are illustrated: The conduction mode with a shallow weld pool at the top and the penetration mode with a black keyhole at the bottom. **(C)** Shows the process of friction stir welding, with the required forces depicted in blue. A close up of the tool shows the non-consumable pin and shoulder.

SMAW uses consumable flux coated stick electrodes which melt during the welding process. The molten electrode adds filler metal to the weld, while the melting of the flux generates a shielding gas which causes bubbles that displace the water from the arc and weld pool. The remaining flux floats to the surface of the weld pool and solidifies into slag, protecting the underlying metal from rapid cooling and environmental influences. This process is illustrated in Figure 3A The slag should be chipped of after welding to increase the overall weld life. This method provides good quality welds and does not require a large amount of equipment. Interestingly, the quality of the weld increases with the depth, as the deposition type switches from short circuit to globular (irregular droplets of molten metal) and the deposition rate increases at a depth of 12.5 m (Mazzaferro and Machado, 2009). However, the quality also depends on the operator skill, since a wrong electrode angle can lead to undesired slag inclusions (Majumdar, 2006).

The tools available for divers are similar to those used on land and consist mainly of an electrode holder, the consumable electrode and a welding machine, providing the necessary power. The latter is typically situated above the surface and connected to the electrode with a long cable, which limits the reachable water depth (Dekker, 2023). A mention of robotic SMAW underwater could not be found, but there are robotic manipulators performing SMAW on land (Lima and Bracarense, 2010).

#### 3.2.2 Flux Cored Arc Welding (FCAW)

FCAW is less dependent on operator skill as it is similar to MIG (metal inert gas) welding and provides high deposition rates. The electrode is composed of a flux cored wire that is incorporated in a welding gun and continuously fed from a spool. As in SMAW the flux solidifies into slag, that must be removed after the welding. A major drawback of this method is the high porosity of the weld and the possibility of burnback, which occurs when the wire welds itself to the tip of the welding gun, so that a new tip needs to be installed. However, special stainless-steel or nickel based wires with halogen free flux compositions have been developed to improve results underwater (Majumdar, 2006).

No special tools for underwater FCAW could be found, so it is assumed that they are similar to those used on land. As it was the case for SMAW, robotic FCAW exists on land, but not yet underwater (ARC Specialities, 2023).

#### 3.2.3 Laser welding

Laser welding produces less heat input than conventional arc welding, which results in a lower susceptibility for cold cracking. Additionally, it has a fast welding speed and results in minimal deformation (Cai et al., 2022). Laser welding can be used in two modes which differ in the amount of power used and the focus, namely, the conduction mode and the keyhole or penetration mode. The conduction mode has a wider focus, resulting in a wide and shallow melting pool, while the penetration mode has a narrow focus, with enough power to cause evaporation in the middle of the melting pool. The hole created by the evaporation absorbs the radiation acting like an optical black body, allowing a deeper penetration of the base material and a better weld quality (Majumdar, 2006) (see Figure 3B). The power needed to generate the weld, is often provided by Nd:YAG lasers, which allow the output through optical fiber, but are quite large (the oscillator can be up to 3.3 × 1.4 × 1.8 m^3^ with optical fiber diameters up to 1 m) (Morita et al., 2006).

A major problem of laser based wet underwater welding is the formation of plasma due to water ionization, which forms a barrier between the laser and the metal, absorbing the laser energy and preventing the weld. This phenomenon occurs at water depths greater than 9 mm, with the laser above the water surface (Cai et al., 2022). Therefore, a stable dry space is necessary for underwater laser welding (Yamashita et al., 2001). This can be achieved by pushing the water away with a shielding gas using special nozzles so that a local cavity is created. Experiments have shown that a gas pressure of 0.2 MPa is needed for successful local cavity welding at a water depth of 20 cm (Han et al., 2021). Since the surrounding pressure increases linearly with depth, it can be expected that the pressure needed for the draining gas also increases linearly. The power needed to generate this pressure would then increase as well, suggesting that local cavity and laser welding become impractical at a certain depth. Nevertheless, Yoda et al. (Yoda et al., 2012) presented a successful example of local cavity laser welding at a depth of 10 m. They developed special tools for the mitigation of stress corrosion cracks in nuclear power plants with pressurized water reactors. These tools are clamped in a tight, cylindrical space and are equipped with a rotating welding head. Additionally, they present a prototype for boiling water reactors, consisting of a robot arm with the welding head as its end effector (Yoda et al., 2012). IHI Cooperation (Tokyo) have developed a remote underwater laser welding robot for nuclear power plants as well, but there is not much information available concerning this robot (Zhu and Jiao, 2011).

#### 3.2.4 Friction welding

Friction welding, as the name implies, uses friction to weld materials together. Usually, one piece is rotated and pressed against the other creating heat at the interface which softens the material. The rotational motion stops, when a suitable temperature is reached and additional pressure is applied to connect the materials. The exact values for parameters like friction pressure, rotational speed, forging pressure and friction and forging time vary depending on the materials used. For a joint between a nickel alloy (In718) and a stainless-steel (SS410), for example, the following optimized parameters have been found: friction and forging pressure 220 MPa, friction time 10 sec, rotational speed 1,300 rpm and forging time 8 s (Anandaraj et al., 2021). This technique is mostly used in production to weld two cylindrical pieces together (Majumdar, 2006).

Friction Stir Welding (FSW), is a special form of friction welding that can be used to join two planar surfaces, which makes it more suitable for repair. Therefore, a rotating tool consisting of a cylindrical shoulder and a concentric smaller diameter pin is used (see Figure 3C). The joint is achieved by inserting the pin into the interface which “stirs” the materials and thereby fuses them together while the shoulder acts as a lid, so that the material does not escape outwards. The probe is then moved along the interface to create the joint, leaving a keyhole when it is retracted at the end of it. This process is mechanized because it requires forces that are too high for manual handling. A joint between two 25 mm thick aluminum plates, for example, would require a downward pushing force of 44 kN along the rotational axis, and a simultaneously applied lateral force of 15 kN. Thinner plates require lower forces but also higher precision, justifying the need for a mechanized process (Colligan, 2009).

Yet another form of friction welding, which is suitable for repairs of thick-walled steel structures with surface or sub-surface cracks is Friction Hydro-Pillar Processing (FHPP) (Bulbring et al., 2013). The process consists of three steps, first a hole is drilled, then a consumable stud is inserted into the hole and rotated while axial pressure is applied. Part of stud then fuses with the base material and the remaining part is cut away in the third step. This can be performed several times along a crack by drilling overlapping holes which is then called stitch welding. The axial forces required for FHPP are lower than those required for FSW and no lateral forces are required. To weld AISI4140 steel, for example, a downward pushing force of 10.5 kN and a rotational speed of 6,000 rpm are required (Kanan et al., 2018; Buzzatti et al., 2015).

Friction welding in general is a solid-state welding process, because the materials are fused without melting. This makes it possible to join dissimilar materials, even a joint between ceramics and aluminum can be achieved (Ahmed et al., 2021). It also reduces many problems associated with molten metal such as volumetric changes, the susceptibility for cold cracking underwater or the need for protective gases, which makes it environmentally friendly and it produces high quality joints (Colligan, 2009).

Since friction welding is a mechanized process, it can not be performed directly by divers. However, this also makes it a suitable process for robotic automation. The project RESURGAM, for example, aims to use FSW together with AI enabled robotics for underwater repairs of ship-hulls (RESURGAM, 2023). During ROBHAZ (Affordable Underwater Robotic Welding System), another research project, a static underwater welding system, which needs to be deployed by a ROV was designed. Therefore, a TRICEPT robot has been adapted to subsea applications and coupled with a welding head for FHPP. The TRICEPT robot was chosen due to its parallel kinematics which allow high forces, precision and repeatability (Meyer et al., 2001).

#### 3.2.5 Challenges for AUVs

As of today commercial wet underwater welding is mostly carried out by divers, which might be due to the difficulties of precise underwater manipulation. Laser welding for nuclear power plants poses an exception, since the hazardous environment calls for alternative solutions. ROVs for underwater laser welding are in development (Zhu and Jiao, 2011), but laser welding requires large oscillators to provide the beam, which are difficult to incorporate in AUVs. Arc-welding might be an alternative since it seems not difficult to transfer robotic arc-welding to the underwater realm. After all, robotic procedures for arc welding already exist. Luo et al. (Luo et al., 2018) have developed a ROV for welding operations in spent fuel pools (SFP) of nuclear power plants. The ROV has 8 thrusters to maneuver and attach itself to the wall of the SFP and a 3-DOF mobile welding platform equipped with a welding camera, a wire feeder and a welding torch (Luo et al., 2018). The main challenge for AUVs however, lies not only in welding but also in detecting and approaching the defect and then performing the manipulation underwater. Another important aspect is the high energy consumption of welding since the AUV has only limited battery power. FSW or FHPP seem to be promising candidates in this regard, but they come with new challenges since the AUV must be firmly attached to the structure in order to apply the high forces needed to produce the weld.

### 3.3 Intervention at oil and gas mining sites

**FIGURE 4:**
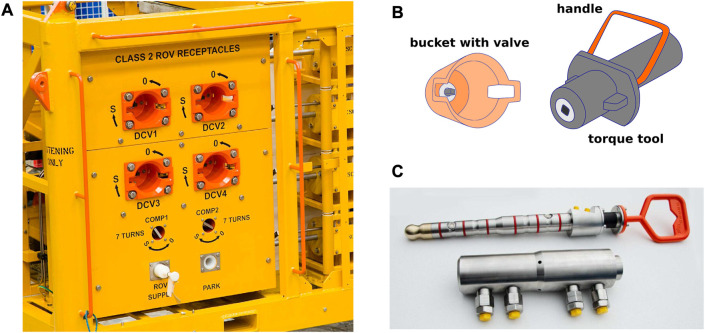
A typical subsea panel for ROV intervention (courtesy of Proserv) is displayed in **(A)**. A torque tool is displayed in **(B)** and a hot-stab in **(C)** (courtesy of DEPRO).

The mining of oil and gas is a complex process, often demanding adjustments of the flow or the injection of protective fluids. This requires the regulation and direction of fluid via a number of valves. Christmas trees, for example, are an assembly of typically eight valves, used to distribute, regulate and monitor the product flow from the well (Bai and Bai, 2010a). The fluid is then further distributed into pipelines or risers through manifolds, which also provide interfaces for the injection of protective fluids or the passing of PIGs (Pipeline Inspection Gauges). In order to control the product flow, the manifolds are equipped with several valves, which can be hydraulically actuated or ROV operated. These are mounted on an intervention panel as shown in Figure 4A. Diver operated valves are not common, since the mining sites are usually at depths that can not be reached by divers (Bai and Bai, 2010b).

Another intervention mechanism at oil and gas mining sites is hot stabbing, which refers to the establishment of a hydraulic connection between a ROV and the subsea system. It is used among others for the hydraulic actuation of valves, the testing of seals and connections and the injection or collection of fluids (Bai and Bai, 2010c).

#### 3.3.1 Valve turning

ROV operated valves are equipped with special interfaces to facilitate ROV intervention. They typically consist of a hollow cylinder, called ROV bucket, which encapsulates a T-shaped handle, or a four edged bolt. The T-handle is usually employed for low torque applications, like ball or needle valves while the four edged bolt is used to transmit high torques used among others for tree valves. The low torque interface, with the T-handle can, for example, be directly be operated with a ROV manipulator, equipped with a claw as an end-effector. For the bolt receptacle, however, a special torque tool is needed, which is depicted in Figure 4B (Bai and Bai, 2010c).

#### 3.3.2 Hot stabbing

Hot stabs used for establishing a hydraulic connection resemble hollow cylinders equipped with several seals around their outer diameter. They typically have a flexible joint at the base, to facilitate the coupling process (see Figure 4C). (Bai and Bai, 2010c)

#### 3.3.3 Challenges for AUVs

A challenge of underwater manipulation that is faced by AUVs and ROVs alike, is the problem of fixation. In order to perform accurate manipulation ROVs are typically equipped with two manipulator arms, one to attach themselves to the structure, preventing the ROV itself from moving and the other to perform the manipulation. However, AUVs are faced with many additional challenges, such as the detection of the correct valve or hot stabbing receptacle, the planning of the end effector motion and it is execution. As a step towards autonomous valve turning, SAAB Seaeye’s Sabertooth AUV was equipped with a TMT torque tool and autonomously navigated towards a subsea panel, where the mating of torque tool and valve was remotely controlled via Sonardyne’s BlueComm free-space optical modem in 2019 (SAAB Seaeye, 2019). Completely autonomous valve turning and hot stabbing has been successfully performed in a pool by a lightweight I-AUV in 2014 (Palomeras et al., 2014). There, a Girona 500 AUV autonomously docked on a mockup control panel, detected a valve with marked edges using a camera and turned it using a 4 DOF manipulator. The receptacle for hot stabbing was detected using a ARToolkit marker. Even though the experiment still needs to be adapted to a real environment where the turbidity of the water might obstruct the view and docking handles for ROVs should be used instead of custom docking panels, this shows that it is possible to perform autonomous manipulation on subsea panels.

## 4 Requirements for autonomy

In the above, the importance of inspection and maintenance of subsea infrastructure was highlighted, and currently employed methods were described. It can be seen, that most of the methods presented here have applications that can be used by ROVs, but currently there are only very few AUVs capable of performing these tasks. However, as seen in Section 1 AUVs have many advantages over ROVs. They require as little human interference as possible and their area of operation is not restricted by the umbilical required by ROVs. The absence of an umbilical also allows for unrestrained movements in confined underwater structures.

**FIGURE 5:**
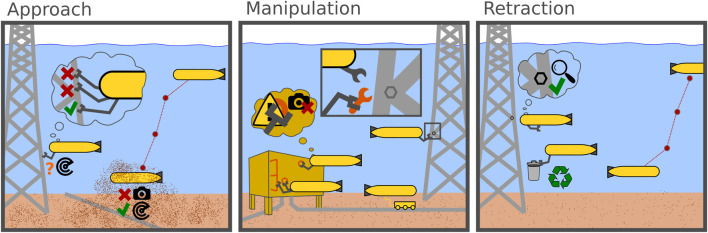
Schematic visualization of the challenges and different possibilities faced by AUVs upon executing a maintenance task.

This section seeks to define capabilities which would be needed in order to perform inspection or manipulation tasks autonomously. Generally speaking, each task can be divided into three subtasks. At first an AUV would have to approach the structure and detect a point of interest, which might be a valve to be turned or a defect to be closely inspected with UT. Then, the inspection or maintenance is performed with the use of tools. This poses a control challenge, as the interaction between vehicle, manipulator and structure needs to be considered and might require docking on the structure in order to transmit forces. The third and last step would be for the AUV to retract to the surface, a subsea residence or the next point of interest (Antonelli, 2018). A visualization of the derived requirements can be seen in Figure 5.

### 4.1 Environmental conditions

Common for all parts of the inspection or manipulation process are the environmental conditions in which the robots have to operate. When talking about marine structures in the energy sector, one can observe that currently most renewable platforms are moored in shallow water environments while oil and gas structures can also be found in depths of up to 2,500 m (Shell Global, 2010). The environmental conditions when operating in deep-sea environments generally do not impose significant disturbances, only low currents are to be expected. Floating offshore wind plants and floating photovoltaic in contrast, are having structures at the ocean floor as well as in the splash zone that require inspection. While the situation at the seafloor can be considered similar to the oil and gas scenario, the splash zone poses additional challenges. Highly turbulent currents around structures as well as waves pose high demands on the control of a robot in order to achieve station keeping, which will be one of the requirements in several phases of the maintenance or inspection tasks (Khalid et al., 2022). The required control strategies for this area of application are a current field of research. The most recent attempts to assess the forces and torques have been reported in (Chellapurath et al., 2022) for a legged robotic system and for an ROV in (Gabl et al., 2021).

### 4.2 Approach

The approach can be further subdivided into coarse waypoint navigation towards the structure and fine sensor-based navigation towards the point of interest. While approaching the structure, an AUV must first master typical navigation related challenges. Since there is no GPS signal, most AUVs navigate by dead reckoning, supported by inertial navigation sensors. Additional sensors are required to detect obstacles and advanced planning is needed to navigate around them. However, since AUVs are already employed for general inspection tasks, these challenges have been discussed extensively (Carreras et al., 2000; Paull et al., 2014; Braginsky and Guterman, 2016) and will not be further investigated here. During the second phase, the AUV must detect the point of interest with its sensors and navigate accordingly. This provides many challenges, depending on the environment. For example, hovering over a structure or fast deceleration might whirl up sand and mud from the seabed, thereby increasing the turbidity in the water and obstructing some sensors. Acoustic sensing technology, such as sonars can provide clear images even in turbid waters, but their capability in the near range, at 1.5 m and lower, is limited (see Section 5.4). Additionally, the point of interest might be surrounded by moorings or jacket-type structures, which requires precise navigation to avoid entanglement or crashes.

Therefore, requirements for the approach, apart from general navigation and obstacle avoidance, would be an accurate close range sensing and the ability to navigate in crowded spaces. For the close range sensing, turbidity invariant sensors would be favorable, but measures to avoid whirling up the seafloor could also be taken. As for the ability to navigate in crowded spaces, a hydrobatic AUV, which can turn about all axes might be a promising approach (Christensen et al., 2022), but there are of course several ways to solve this challenge, depending on the specific use case.

### 4.3 Manipulation

Once the AUV has found the point of interest, the original task must be performed. For most of the tasks, a stable distance to the surface is required, which can be achieved in two ways. The traditional method, currently employed by most ROVs, would mean for the AUV to fix itself to the structure and then perform the task (Meyer et al., 2001; Palomeras et al., 2014; Sivčev et al., 2018; Bae et al., 2022), whereas an alternative would be to perform the task while freely floating in the water column. Fixed manipulation is easier to control, since the movement of the end-effector relative to the vehicle is also relative to the world, it is potentially more energy efficient and it can provide larger forces than free floating manipulation (Bae et al., 2019). This makes it favorable for heavy tasks, such as cleaning, welding or valve turning. Free floating manipulation, per contra, is a highly researched alternative (Youakim et al., 2017; Ding et al., 2021; Topini et al., 2021; Lv et al., 2022) that could be used for tasks requiring low forces such as inspection tasks or hot-stabbing operations. However, free-floating is less mature than fixed manipulation. As of now, free floating valve-turning has been successfully performed in test basins (Youakim et al., 2017) and picking up objects has been demonstrated in shallow seawater (Simetti et al., 2014). However, to the best of the authors’ knowledge, there are no industrial applications of free floating manipulation. The main advantage of free floating manipulation is the omitting of the docking process, which is necessary for fixed manipulation. This process is in itself a complicated task since the AUV must first determine where to attach itself, therefore determining suitable fixation points, that are strong enough and from where all pints of interest can be reached. Then the fixation itself must be performed for which the AUV might need an extra manipulator arm, or a special docking device, depending on the structure and an advanced control mechanism, for docking with minimal impact.

Another possibility, that allows the performance of heavy tasks, would be the deployment of small crawlers, as it is already performed by ROVs. Thereby, the AUV does not need to dock itself on the structure, but simply deploys a crawler which will attach itself and carry out the tasks. Of course, the docking problem still exists, but the crawler can be purpose-build for a specific task and structure, thereby facilitating the process of fixation. Additionally, crawlers can easily hold a stable distance to the structure, and they can incorporate several tools at once, making them a versatile option. Furthermore, small inspection crawlers, dedicated for the use by ROVs already exist and often have an in build automatic scanning procedure, which might facilitate the incorporation in AUVs (Jeppesen et al., 2005; Reber et al., 2016). This requires a compartment to store the crawler, deploy it and handle the connecting fiber autonomously which poses additional soft- and hardware challenges.

Even though crawlers seem to be a good solution, they can not be deployed on structures with complex geometries. The inspection of corner or T-shaped joints, for example, would be difficult to achieve with crawlers. Therefore, other tools are employed as well. Looking through the methods explained in Section 2 and Section 3, many different tools were used, each specialized for its task. However, they can be divided into two categories.

• Manipulator or hand held tools equipped with a handle (e.g., ACFM or diver performed welding) and
• Fixed tools, mounted on the manipulator or the case of the ROV itself (e.g., UT, or cleaning).

Manipulator held tools enable a more versatile use of the manipulator compared with fixed tools since different tools can be used by the same end-effector. However, they also come with additional challenges since the AUV needs a compartment to store these tools and the manipulator motion must be carefully planned to pick the right tool from the storage in the correct position. Furthermore, there is a risk of losing the tools, due to failed grasping, so they should be attached to the AUV with a small rope. Tools which require actuation need to be connected to the AUVs power supply. However, depending on the amount of energy consumed by the tool, strong batteries would be needed. This is a major problem of cleaning AUVs, Mai et al. (Mai et al., 2022) even deemed “onboard battery power infeasible”. Another intricacy arises due to the changed mass distribution when picking up tools or other objects. If unaccounted for, the change in mass distribution, alters the vehicle orientation and must, therefore, be handled by the control mechanism.

On top of the challenges imposed by the chosen tools and manipulation method, the control of the end-effector provides a challenge of its own. As mentioned in Section 4.2, sensor based navigation can be difficult in close ranges, which is additionally complicated by the fact, that the moving end-effector sometimes obstructs the sensors. This might be solved by placing the sensors such that they can not be obstructed, either directly on the end-effector or further to the side, where they are less likely obstructed. Additionally, sensitive force-feedback control mechanisms should be implemented, to avoid damaging the structure or tools as well as to receive tactile feedback while performing the task. A promising field of research in this regard are also soft robotic solutions, where especially in the manipulation part of inspection and maintenance task interesting solutions for form-closed manipulation might be achievable. As this domain is mostly still in research, the interested reader is referred to (Aracri et al., 2021) for further reading. Finally, the motion of the vehicle is much slower than that of the manipulator. This is especially important in free-floating manipulation, since the controller of vehicle and arms should work at different frequencies even though they are connected. From these challenges, requirements for sensors, software and hardware can be derived, which are listed below:

Sensorwise:

• Using reliable close range sensors
• Placing the sensors in a way they won’t be obstructed by the end-effector

Softwarewise:

• Planning of the trajectory of the end-effector(s), with or without picking up tools
• Successful execution of the trajectory, including the handling of unexpected obstacles
• Controlling the vehicle to keep a stable distance to the structure
• Using force-feedback control to avoid damaging the structure

Hardwarewise:

• Providing a large battery capacity
• Providing a hydrodynamic storeroom for tools or crawler
• Mechanism for autonomous fiber handling

### 4.4 Retraction

Once the task is performed, some post-processing might be needed depending on the operation. Especially after maintenance tasks such as cleaning or welding, some quality control measures should be taken to ensure the optimal performance of the task. For representational purposes, before and after pictures could be taken, and welding seams could be examined with acoustic or electromagnetic tools to make sure that there are no cracks remaining. Online interpretation of this data would be desirable in order to achieve optimal results. If, for example, pictures taken after cleaning were examined for remaining stains, the cleaning could be resumed in these parts until all stains are removed. However, the online interpretation of data would require a substantial amount of computational power, posing an additional challenge for intervention AUVs.

Another aspect of post-processing might be to tidy up the area in order to make sure that nothing is left behind. This includes the recuperation of tools and crawlers, which must be stored safely inside the AUV before it starts the journey home. But it can also extend to the gathering of loose paint or marine species that came off during cleaning. This is especially important while cleaning ship hulls, since they travel through different ecosystems and might carry invasive species (Floerl et al., 2010).

After the post-processing the AUV might need to detach itself from the structure and navigate to the next point of interest, whether that is another task or the surface. This is however a navigation task that is already addressed by current AUVs (as explained in Section 4.2), and will therefore not be discussed any further.

## 5 Current technology

Now, that the capabilities which are necessary for autonomous inspection and manipulation are derived, the state of the art of current AUV and manipulation technology will be presented. We will discuss how well the requirements for autonomous manipulation are already met and gaps between technology and requirements will be highlighted. This discussion will be based on a qualitative analysis, since quantitative data is rarely to be found. Further quantitative insights require reliable benchmark tests, which would be of high benefit for the community.

Even though intervention AUVs have not yet reached the technological readiness to be widely employed by the industry, there are some prototypes developed in a research context. In addition, there are several ROVs that have been equipped with some intelligence in order to facilitate the operation. This section will first focus on these existing platforms presenting the capabilities of some commercial UUVs equipped with manipulators followed by some intervention AUVs developed in a research context as well as an unconventional solution for underwater manipulation in the form of Underwater Swimming Manipulators (USM).

As a foundation for autonomy, environment perception especially on the near range below 1.5 m distance to objects is crucial for autonomous closed range inspection and maintenance task, as it can be expected that this distance will be the maximum range of a manipulator arm mounted to an underwater robot. Thus, we will present the current capabilities and limits of sensing technology in this range and discuss potential further developments.

### 5.1 Commercial intervention AUVs/ROVs

#### 5.1.1 Aquanaut

Aquanaut has been developed by Houston Mechatronics Inc. (now called Nauticus Robotics) as a minimally supervised unmanned underwater vehicle (UUV) and was presented in May 2019. According to Manley et al. (Manley et al., 2019), it can dive deeper than 3,000 m and combines the streamlined shape of an AUV with the stable box shape of a ROV by transforming from one shape into the other when needed. For the transformation, the hull opens vertically and reveals two electric, 8-DOF manipulators, as well as additional thrusters for better maneuverability. Further specifications can be found in the IEEE robots-guide (IEEE Robots, 2023).

As stated in Manley et al. (Manley et al., 2019), the head, containing machine vision cameras and other sensors, can rotate so that the sensors, which pointed at the bottom in AUV-mode, are directed to the front of the vehicle where the workspace of the two arms is located. Aquanaut then acts based on a 3D model of its surroundings that is either generated by on board sensors, such as a custom 3D structured light sensor, or given by the operators before deployment. According to Manley et al. (Manley et al., 2019) the robot is operated without a tether, but high level commands can be sent via Ultra-Short BaseLine (USBL).

Due to its dual arm configuration Aquanaut is able to perform both fixed and free-floating manipulation using hand-held tools. In a video, posted by Nauticus Robotics in 2020, Aquanaut can be seen in a pool, unplugging and plugging a mock-up hot-stab into a mock-up panel. Unplugging is done in fixed manipulation mode, while holding a handle with one hand, while plugging is done freely floating (Robotics, 2020).

Nauticus Robotics has developed a MK2 version of Aquanaut in 2023 (Offshore, 2023), reducing the DOFs by omitting the transformation mechanism, and using 6-DOF arms instead of 7-DOF. The new vehicle is larger than the prototype, weighing 4,200 kg in air. More detailed specifications can be found in (Nauticus robotics, 2023).

#### 5.1.2 Kawasaki SPICE

SPICE stands for Subsea Precise Inspector with Close Eyes and was developed by Kawasaki for autonomous close range pipeline inspection. Therefore, the AUV is equipped with a special manipulator, resembling a long stick that is folded parallel to the vehicle but can be flapped downwards to drag a wheel-equipped end-effector (EE) over the pipeline. The vehicle was introduced in the market in 2021 (Sakaue et al., 2020).

According to the Kawasaki group, SPICE can achieve up to 4 knots using 5 thrusters, one for main propulsion and two for vertical and horizontal control each. The cruising speed of 2 knots, together with 8 h battery capacity sums up to a range of 20 km. As reported by Kawasaki, the elongated manipulator can be controlled in rotation around all three axes at the base and the tip, making it a 6-DOF arm. The end-effector is a skid with four wheels on which inspection sensors, such as cameras or cathodic protection sensors are installed, thereby avoiding the risk of occlusion. Kawasaki points out, that the arm is automatically raised when an obstacle is detected and lowered again after it is passed (Kawasaki Group Channel, 2020; Sakaue et al., 2020).

The vehicle is launched together with a docking station, which, as the Kawasaki Group explains, remains underwater so that the vehicle can return autonomously, charge and upload data, before starting another mission. They state, that USBL and optical communication is used for the docking process as well as a guiding rail, to which the AUV attaches before docking completely (Sakaue et al., 2020).

According to Energy Voice (Cresswell, 2020) SPICE proved its capabilities during field trials in 2020, where a mock-up pipeline fitted with obstacles was tracked, and the arm was tested at various speeds. In total, the AUV dived for more than 50 h, driving 16 km, 14  of which the mock-up pipeline was tracked. The docking process has also been successfully tested.

#### 5.1.3 Sabertooth

The Seaeye Sabertooth is a hybrid between ROV and AUV developed by SAAB Seaeye in 2011, which was designed to perform long time missions around Subsea Production Systems (SPS). Additionally, a subsea docking station and a communication network were developed, so that, according to Johansson et al. (Johansson et al., 2011), the AUV can remain at the seabed for up to 6 months, without requiring service.

Saab Seaeye states, that the Sabertooth has a depth rating of 500 m which can be extended to 3,000 m when using a double hull. They claim, that the vehicle, equipped with six thrusters, achieves a forward speed of 5 knots and can roll up to 90° (Johansson et al., 2011). For manipulation the Sabertooth is equipped with a directly attached torque tool, which can be rotated 90° to the side, in order to access vertical valves. While connected to the valve, the flexible joint allows some angular movement of the Sabertooth, thereby reducing the bending load on the valve. This is additionally reduced by the neutral buoyancy of the vehicle. Saab Seeye states, that in the future, a tool skid can be docked to the AUV, which will allow the use of different tools (Johansson et al., 2011).

For environment perception the company reports, that the hybrid AUV/ROV is equipped with several (Palomeras et al., 2014; Simetti et al., 2018; Christensen et al., 2022) fixed cameras, sonars and a hydrophone which is used as a self monitoring tool. While navigating in the SPS, the Sabertooth can use passive landmarks in the form of sonar reflectors or radio frequency identification tags. Electromagnetic waves are used for communication, which allows a bandwidth of 10 Mb in proximity of the docking station and around 100 kb otherwise. An interesting feature, explained by Johansson et al. (Johansson et al., 2011) is the short (10–15 m) tether which is attached to the communication antenna, this allows the AUV to leave the antenna hooked to the docking station, enabling short range missions with high bandwidth (Johansson et al., 2010; Johansson et al., 2011).

As reported by Johansson et al. (Johansson et al., 2011), there are three different modes in which the Sabertooth can be operated. The autonomous mode allows the execution of a specific task, such as a transit. The autonomous capabilities include among others the following of waypoints, obstacle avoidance, station keeping and auto heading or altitude. The operator-assisted mode, allows the control via step by step high level commands, whose execution is verified by video or sonar data. Manual operation is also possible, where the pilot flies the Sabertooth with a joystick, all the while being assisted by the IMU and DVL, to account for the low bandwidth (Johansson et al., 2011).

The Sabertooth was tested in a Lake in Sweden, where, according to Saab Seaeye, underwater docking was demonstrated and the mating between torque tool and valve panel was performed. During these tests, the vehicle was manually controlled using the operator-assisted mode (SAAB Seaeye, 2019). Additionally, two Sabertooths were employed to search for the Wreck of the Endurance in the Antarctica, which they found at a depth of 3,008 m, proving their capability to reach great depths (Saab, 2022).

### 5.2 Intervention AUVs in research context

#### 5.2.1 Ocean One

Ocean One is a robotic avatar that has been developed by Stanford University in 2016, together with KAUST’s Red Sea Research Center and MEKA Robotics. It is meant to perform tasks typically performed by human divers including the assembly of structures and the delicate handling of irregular shaped objects. According to Brantner et al. (Brantner and Khatib, 2021) The system works as an ROV and is operated through an Ethernet cable, that connects it to the surface, the depth rating lies at 200 m. However, it provides some autonomy in performing predefined tasks, only requiring human intervention when it fails (Brantner and Khatib, 2021). The robot has approximately human dimensions and the front part is shaped like a human torso with head, shoulders and arms, while the rear contains a total of 8 thrusters, four of which control the yaw and horizontal translation and the other four control the roll, pitch and vertical translation (Khatib et al., 2016; Brantner and Khatib, 2021).

In order to perform human-like intervention, Ocean One is equipped with two 7-DOF arms that are driven by series elastic actuators (Pratt and Williamson, 1995) and equipped with torque-controlled joints in order to permit compliant, force controlled motion. As stated in Stuart et al. (Stuart et al., 2017) The hands have three under-actuated tendon driven fingers driven by a single actuator, each composed of three segments. Six-axis force torque sensors are incorporated at the wrist. This allows compliant and adaptive grasping as the fingers close around an object until it is completely wrapped. According to Brantner et al. (Brantner and Khatib, 2021) several cameras are used as close range sensors, two in the head, which allow for stereo vision and one wide angle camera in the chest. This amount of cameras helps to reduce obstruction of the field of view. For further amelioration, Brantner et al. (Brantner and Khatib, 2021) state that tilt and pan motion of the head is planned.

In order to control Ocean One, a novel whole-body control was implemented, as explained in Brantner et al. (Brantner and Khatib, 2021), which combines the control of the fast reacting arm- and the slower reacting body-posture. This provides functional autonomy so that the 20 DOFs of the Robot (7 for each arm and 6 for the body posture) can be controlled by specifying the pose of the two hands and allows fast dynamic responses since the movement of the manipulators buffers the slower body movement. The control is based on a task hierarchy, where constraints, such as joint limit avoidance, self-collision avoidance and obstacle avoidance, supersede the manipulation task, which in turn supersedes posture tasks. The posture tasks are based on maintaining a preferred body posture relative to both wrists and a preferred arm posture which optimizes the hand poses for the respective task (Brantner and Khatib, 2021).

Brantner et al. (Brantner and Khatib, 2021) mention, that besides fully remotely controlled operation, Ocean One provides a semi-autonomous operation mode based on predefined skills. This was demonstrated in a pool by inserting a flagpole in a hole. In this case the pilot uses constrained haptic interaction, only conrolling the horizontal motion of the robot, while all other motions are controlled by the skill.

#### 5.2.2 Girona 500

The AUV Girona 500 was developed at the underwater robotics laboratory of the university of Girona in Spain in 2012 and has since been the platform for many projects. According to Ribas et al. (Ribas et al., 2012) It was designed to be compact while having a large payload capacity. As a compromise between a streamlined shape and a passive stability with separated center of mass and buoyancy, the vehicle is composed of three torpedo shaped hulls held together by an aluminum frame, two light ones at the top and a heavy one containing the battery at the bottom (Ribas et al., 2012).

As the name suggests, Girona 500 has a depth rating of 500 m. In order to configure the platform to the task at hand, Ribas et al. (Ribas et al., 2012) state, that the AUV can accommodate between 3 and 8 thrusters, though four is the standard configuration in which two thrusters are used for heave and pitch stability and the other two for surge and yaw (Ribas et al., 2012).

The Girona 500 AUV has proven its manipulation capabilities in various projects with different manipulators and with ongoing research in the challenging domain of handling manipulation tasks underwater. In the TRIDENT project, free-floating autonomous object grasping was successfully performed using a 7-DOF arm and a three fingered dexterous hand in pool- and sea-trials. Camera occlusion was actively avoided using the redundant DOFs and the vehicle odometry was used for object pose estimation when occlusion was unavoidable (Simetti et al., 2014). Additionally, the hand was equipped with tactile sensing, allowing to receive haptic feedback from the object (Sanz et al., 2012). The combined operation of vehicle and manipulator was managed using multirate control and task priority based algorithms. Another approach for control was used by Youakim et al. (Youakim et al., 2017), which performed free-floating valve-turning and hot-stabbing with a 4-dof arm using MoveIt, a ROS package that can plan and execute motions (Chitta et al., 2012). This was tested in a laboratory environment. During the TWINBOT project (Pi et al., 2021), two vehicles were used to cooperatively pick, transport and place an object in a pool. To avoid occlusion, cameras were placed in the palm of the end-effector.

#### 5.2.3 Cuttlefish

The Cuttlefish is a hydrobatic intervention AUV developed by the Robotics Innovation Center of the German Research Center for Artificial Intelligence (DFKI) in Bremen. Hydrobatic vehicles are typically able to take arbitrary poses in the water, and according to Christensen et al. (Christensen et al., 2022) the Cuttlefish can change its center of mass and buoyancy to transit between a more stable or more agile configuration. A special docking interface is also included to allow fast data transmission and human intervention once docked to a subsea panel. These capabilities have been successfully tested in a saltwater basin (Christensen et al., 2022).

The AUV has a flat, rectangular shape. It is equipped with 8 low rpm ring thrusters each generating up to 500 N, two of which are placed in each corner. As an additional DOF, the two battery compartments are linearly actuated to shift the center of gravity. The diving depth of the whole system lies at 300 m (Christensen et al., 2022; DFKI, 2023).

For intervention purposes, Christensen et al. (Christensen et al., 2022) state, that the Cuttlefish is equipped with two in-house developed manipulator arms. The docking arm has 4-DOF and a gripper, in which a WI-FI antenna is integrated. This arm attaches to a spherical docking device on the asset, in which another antenna is installed, permitting very close range wireless communication (Wirtz et al., 2012) underwater at 70 Mbit/s. The manipulation arm has 6-DOF to which different tools weighing up to 7 kg can be mounted. Force sensing was not yet implemented in the arms in 2022 but is planned. To perform manipulation, Christensen et al. (Christensen et al., 2022) explain, that the vehicle pitches 90 deg, so that the bottom faces to the asset in front. This causes the DVL to lose bottom lock, which is why a stereo camera is attached to perform visual navigation. The stereo vision is chosen to avoid obstructions and provides the possibility of 3D measurements, which could be compared to CAD drawings. A second smaller DVL is attached to the rear of the AUV which becomes the bottom in manipulation mode (Christensen et al., 2022).

Since the vehicle can achieve pitch angles of 90°, quaternions and rotation matrices are used for the representation of its pose. The vehicle is controlled using a cascaded PID controller, the first layer controls the position and orientation and the second layer consists of six independent PID controllers for the twist. Model-based feedback linearization is then used to compensate for hydrodynamic effects and the control allocation takes the thruster limitations into account, becoming an optimization problem formulated as mixed integer quadratic programming. The manipulator arms are controlled via ROS using MoveIt. Since the complete kinematic chain of both arms and the vehicle can be specified in MoveIt, self collision is avoided and planning and trajectory execution can happen for both arms in parallel (Christensen et al., 2022).

#### 5.2.4 Further hydrobatic vehicles

Other hydrobatic robots include MARINS (Maritime Research Institute Netherlands) modular AUV (mAUV) (Cozijn et al., 2019) and the SAM AUV developed by the Swedish Maritime Robotics Center (SMaRC) (Bhat et al., 2022). In contrast to the Cuttlefish, both have a streamlined torpedo shape and are not intended for use in intervention tasks. According to Cozijn et al. (Cozijn et al., 2019), the mAUV is highly overactuated using 4 stern thrusters and two vertical and horizontal thrusters at the bow and stern. Additionally, a moveable mass and a buoyancy trim system is integrated. It is controlled using a state feedback based PID controller based on proprioceptive sensors (De Kruif et al., 2019). The AUV SAM, on the other hand, is underactuated having only one thruster with two counter-rotating blades at the stern, and a moveable mass, rotating weights and a buoyancy trim system for roll, pitch, and depth control as stated by Bhat et al. (Bhat et al., 2022). The control is also based on state feedback but uses nonlinear Model Predictive Control (MPC) for optimization (Bhat et al., 2022).

### 5.3 Underwater swimming manipulators

A different approach to the challenge of underwater manipulation are underwater swimming manipulators (USM). These have a snake like shape, with joints allowing to bend the body, but are also equipped with thrusters, for transit and hovering capabilities. In 2016, the company Eelume AS build a first prototype called Eely. According to Liljeback and Mills (Liljeback and Mills, 2017) Eely has a modular configuration, consisting of 2-DOF joint modules which allow motion in pitch and yaw, lateral and longitudinal thruster modules and other modules containing sensors or a tether interface (Liljeback and Mills, 2017).

Liljeback and Mills (Liljeback and Mills, 2017) state that, the vehicle has a length of 3.37 m a diameter of 0.18 m, a total weight of 85.6 kg and a depth rating of 150 m in the final configuration. The kinematic chain consists of 9 joints and 8 links which alternately address pitch or yaw and the vehicle is actuated by 7 thrusters located in the middle of the robot, as explained by Borlaug et al. (Borlaug et al., 2020). Two for surge and one for heave are placed in the fifth link and two for heave and yaw in the third and seventh link each. This prototype was tested in a dry-dock in Trondheim, where it moved between underwater structures (Liljeback and Mills, 2017; Borlaug et al., 2020).

For the control of articulated intervention AUVs (AIAUV), to which the USMs belong, a combined kinematic and dynamic control was proposed by Borlaug et al. (Borlaug et al., 2020). They employed a singularity robust multiple task priority framework (SRMTPF) together with a sliding mode controller (SMC). Any SMC could be used, as long as it ensured a convergence of the velocity vector to the velocity reference vector provided by the SRMTPF. This avoids the assumption of perfect dynamic control and proved robust in comparison to a PID and feedback linearization control (Borlaug et al., 2020).

According to Eelume AS (Eelume, 2023), the next-generation, Eelume 2020, is enhanced with a sensor and communication module, and the two thruster-modules are merged into one, with two lateral thrusters for yaw control and two longitudinal thrusters tilted by 45° for heave and surge. Since it is a modular system, several tools such as grippers, cleaning or cutting tools can be attached everywhere along the body making dual arm intervention possible by attaching grippers to the front and back of the vehicle (Ma et al., 2022; Eelume, 2023).

### 5.4 Environment perception

As can be observed from the current system technology of underwater robots for maintenance and repair tasks, most of the systems rely at least partially on human support during their missions. When attempting to perform fully autonomous intervention, all stages of the operation have to be perceived and processed by the robot. An especially crucial part for the success of the operation is the environmental perception in the area of operation in order to identify the correct operation location. Derived from current underwater manipulator technology, it can be assumed that interaction tasks with underwater structures will happen at 1.5 m distance and lower from the asset structure.

However, the exteroceptive sensing technology currently employed on most autonomous underwater robots is more suited for far range sensing in distances beginning at 5 m and more. Furthermore, as the environment perception addressed in the context of underwater maintenance and repair requires three-dimensional sensor feedback, this kind of sensing technology will be investigated further.

**FIGURE 6:**
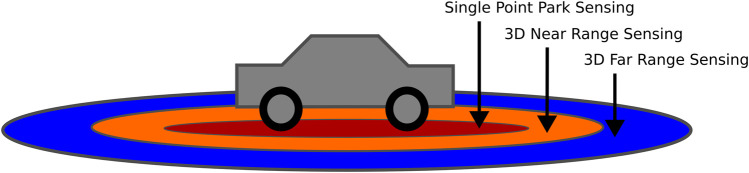
This schematic shows how autonomous cars perceive their environment based on multimodal sensor input.

Comparing the application scenario for environment perception, similarities can be found in the autonomous driving sector where different sensing modalities cover certain levels of distances for various ways of approaching objects (see Figure 6).

In the very near range between 1 cm and 1 m, ultrasonic altimeter sensors can be compared to the frequently used ultrasonic sensors for obstacle sensing in autonomous driving. The sensing range here starts a 1 cm underwater and is offered in millimeter resolution (Impact Subsea, 2023a). For the medium range sensing range between 0.5 m and 5 m a survey on the market of commercially available underwater sensing devices yields, that LiDAR technology as offered by 3D at depth (3D at depth, 2023) starts to operate at 2 m distance from objects and higher, while the Voyis camera laser system works at 1.2 m minimum distance (VOYIS, 2023). The Kraken Seavision Camera-Laser system states to have submillimeter resolution in distances of 1 m to the object (KRAKEN ROBOTICS INC, 2012). As these technologies all operate based on vision sensors, it is worth having a look at sonar technology, where imaging sonars like the IS360HD are offering working distances starting at 0.45 m with a resolution of 2.5 mm (Impact Subsea, 2023b). Further scientific work describes initial results to develop an underwater time of flight camera (Mack et al., 2019), which seems to offer distance of operation in the cm range.

As can be observed from the current technology in underwater robots, occlusion of the workspace is another challenge that needs to be addressed when performing non-destructive testing operations. Here, especially tactile sensing (Cong et al., 2021) or a set of cameras arranged at different angles observing the area of operation are potential solutions to these challenges.

### 5.5 Conclusion

The intervention UUVs presented above, proved successful in retrieving objects or performing valve turning. As described in Section 4 these tasks come with challenges, which were partly addressed by the vehicles. Below, a summary of these challenges as well as their proposed solutions is listed:

Close range sensing:

• Close range sensing and navigation based on cameras (used by all presented vehicles)

Avoiding obstruction:

• Using stereo vision or multiple cameras to avoid obstruction. (Ocean One, Cuttlefish, Sabertooth)
• Actively avoiding obstruction before final grasp using redundant DOFs (Girona 500)
• Placement of sensors in the palm, so that the end-effector can not obstruct them. (Eelume, Spice)

Force feedback:

• Incorporating force and torque sensors in the wrist, which enables force feedback control. (Aquanaut, Ocean One)
• Using tactile sensors in the fingers, which allows force controlled grasping. (Girona 500)
• Equipping the joints with compliant actuators, to use structural compliance for soft contact. (Ocean One)

Fixation when using fixed manipulation:

• using a second manipulator to grasp a handle or special docking device (used by all presented vehicles)

Control of arm and vehicle:

• Using task priority control to handle multiple tasks with different levels of importance. (Ocean One, Girona 500)
• Combining the control of the vehicle and arm and considering the different control rates. (Ocean One, Girona 500)
• Combining the control without considering different speeds (Girona 500)
• Controlling the manipulator with MoveIt, which avoids self collision. (Girona 500, Cuttlefish)

## 6 Discussion

### 6.1 Persisting challenges

It can be seen above, that some requirements for autonomous intervention, which were derived in Section 4, are already met by the current technology. However, most of the vehicles presented above were remote controlled or semi autonomous, showing that true autonomous underwater manipulation is still a challenge. In the following, the major issues that make autonomous intervention difficult are highlighted.

The characteristic that distinguishes AUVs from other UUVs is their capability of decision-making. However, this may be the most complicated part. Considering underwater intervention, decision-making extends to detection the correct point of interest, finding an orientation that maximizes the workspace of the manipulator, planning the manipulation such that the right amount of force is exerted, and monitoring the progress to allow for corrections and high quality results. So far, these decisions are made by humans only. Fully autonomous valve turning, for example, which has been successfully performed in laboratory environments, has, to the best of the authors’ knowledge, not yet been performed on real manifolds.

In order to support the decision-making, a precise understanding of the complete working space is needed, as well as good self-perception. Therefore, reliable close range sensing is indispensable. This is related to the problem of occlusion, which is currently solved by using multiple cameras and/or tactile sensors (see Section 5.5). However, how to best arrange these sensors to gain a complete workspace overview regardless of the manipulator position and how to combine multimodal input remains unaddressed. While executing a task, continuous quality control is desirable, e.g., to detect if a contact is stable. For this, decent perception of the working space as well as reliable post-processing is required.

To find the point of interest, a precise localization mechanism is needed. Sabertooth used a promising landmark-based navigation, but they only achieved a precision of <2 m, which is not sufficient for autonomous navigation (Johansson et al., 2011) in proximity to underwater structures. Therefore, precise navigation near the point of interest remains an open challenge.

Once the point of interest is detected, the AUV should position itself in a way that maximizes the workspace of the manipulator. Using *a priori* known information on the structure, suitable handles and postures can be determined in advance to facilitate the process. However, determining proper handles autonomously is a challenge yet to be addressed.

Depending on the task, different tools might be needed. So, if several operations are to be addressed, the tools must be changed in between. As of now, most intervention UUVs, excluding working class ROVs, are build for a single task only and can not change their tools. Working class ROVs, often have a tool skid, from which they pick tools with a gripper, but using this principle comes with additional issues for AUVs as they need to determine a good grasping pose (see Section 4). Other possibilities include a revolver like tool drum at the end-effector or a docking interface, that allows to autonomously attach and detach tools. Another challenge that comes with the use of tools is their power consumption. Depending on the job, the battery of an AUV can not handle the required power (Mai et al., 2022). This could be solved by using tools that are externally powered, for example, connecting to a subsea docking station, or equipped with their own battery. However, these are all issues that still persist.

During cleaning, another challenge arises, that has not been addressed so far: The collection of removed material. This includes potentially invasive species, when cleaning ship hulls but also ablated paint or coatings, that might be dangerous to the environment. In order to avoid these hazards, removed material could be collected in a net or sucked away by something like an underwater vacuum cleaner.

### 6.2 Conclusion and outlook

In this survey, the inspection and maintenance tasks required by the industry and the currently employed tooling were described and the current state of underwater robots was presented. From this, major challenges which need to be addressed in development were derived.

In order to sustain the integrity of structures, regular inspection and maintenance is needed. This includes thickness and corrosion measurements, as well as the detection of cracks and the measurement of cathodic protection. Most inspection methods are non-destructive, but require close contact for which the structure must be cleaned. Other required maintenance includes the welding of cracks and the intervention on SPS consisting of valve turning and hot stabbing.

Currently, close range inspection tasks are mostly performed by human divers and ROVs, with the exception of the SPICE AUV, while general inspection is also performed by AUVs. The tools are designed accordingly, meaning that most of them are handheld and require an external power source. Some inspection tools are incorporated in scanners, which can be deployed by ROVs and firmly attach to the structure to be inspected (see Section 2). Nevertheless, first attempts towards autonomous underwater close range inspection are demonstrated, as shown by the SPICE AUV.

Maintenance tasks on the other hand require more interaction with the structure, needing advanced force-feedback control and high precision for some tasks, making it more demanding for AUVs. Therefore, intervention AUVs have yet to conquer the market. As of now, cleaning is performed by ROVs and humans alike, SPS intervention is done using ROVs only, due to the great depths, and underwater welding is mostly executed by human divers even though some specialized ROVs exist. There exist underwater laser welding robots specialized for nuclear power plants (see Section 3.2.3) and there is ongoing research regarding underwater friction welding robots (see Section 3.2.4). Cleaning robots are often highly specialized, having strong adhesion mechanisms and several fixed cleaning tools, but there exist also handheld water jetting guns for divers. For SPS intervention handheld torque-tools or hot-stabs are used as well as fixed ones, and for welding only diver related handheld tools exist (see Section 3).

It can be seen, that the tools are optimized for the use by divers or ROVs, and therefore, some development might be needed in order to incorporate them into AUVs. This is especially the case for heavy-duty cleaning or welding equipment that requires a large amount of power. Scanners might be easier to integrate since they already provide autonomous inspection capabilities, however an autonomous transport and deploy mechanism is needed.

There are already some semi-autonomous and AUV/ROV hybrid vehicles that are capable of underwater intervention and even some examples of successful autonomous valve turning in a controlled environment (see Section 5). Ocean One, for example, is able to execute preprogrammed skills, which allow the grasping and turning of valve. However, human supervision is still required, since the robot can not react to unforeseen events. AUV/ROV hybrids, like Sabertooth can autonomously transit to the point of interest, but require human control upon arriving. The communication is relayed to an onshore facility over a subsea docking station, omitting the need for a surface vessel. However, completely autonomous systems for underwater manipulation have not yet been deployed commercially.

In Section 6.1 the decision-making required for autonomous intervention was deemed one of the most difficult challenges yet to be addressed. And an important question still remains regarding the level of autonomy required. A low level would mean to preprogram a mission based on known information, similar to the skills performed by Ocean One but more extensive. Such a mission could include the best posture for the task execution and marked fixation points which provide a stable hold while maximizing the possible workspace. This approach has a low level of complexity, but it can not react well to disturbances, such as a broken hold. A high level of autonomy would mean to specify only the point of interest and let the vehicle determine good attitudes, fixture points and required forces to execute the task. This approach is more stable against disturbances, but has a much higher level of complexity, requires a lot of computational power and might lead to unexpected behavior. A suitable approach might lie somewhere in between these two extremes, but also depends on the task. Inspection tasks, for example, might be suited for a mission based approach which is of course easier to implement and can already be seen in the SPICE. Manipulation tasks on the other hand might require more flexibility.

Another aspect of underwater intervention is the existing infrastructure to be inspected or repaired. Since SPS intervention is currently done by ROVs, suitable handles for fixation are already present. To facilitate autonomous intervention, however, further interfaces might be needed. The installation of passive landmarks such as sonar reflectors, for example, can guide the AUV to the point of interest with a high precision. Frequently visited points, such as important valves could be labelled with markers for different sensing modalities to enable a fast detection. Furthermore, the installation of underwater power cables with accessible interfaces would be beneficial whenever heavy-duty tooling, such as welding or cleaning equipment is required.

To sum it up, the characteristics of the perfect intervention AUV are difficult to determine. They certainly depend on the required tasks, but also on the environment and level of autonomy desired. A quantitaive analysis in this field is therefore difficult to perform, but we sought to provide a qualitative insight on the different challenges and possible solutions for underwater inspection and manipulation. To gain quantitative data, benchmark tests similar to the RAMI competition (Ferri et al., 2016) could be envisioned, but these have yet to be designed. The current vehicles that come closest to intervention AUVs range form stable under-actuated platforms with one manipulation arm (Girona 500) over highly maneuverable hydrobatic vehicles equipped with two arms (Cuttlefish) to snakelike USMs that are highly configurable and can attach tools to their front and rear end (Eelume). These many shapes highlight the diversity of underwater vehicles and the many directions in which the development of intervention AUVs expands. The overview given in this paper can by no means encompass all the current development, but it seeks to highlight links between the current technology and the industrial requirements and to serve as inspiration for further development.
